# Electronic Transport Mechanism for Schottky Diodes Formed by Au/HVPE a-Plane GaN Templates Grown via In Situ GaN Nanodot Formation

**DOI:** 10.3390/nano8060397

**Published:** 2018-06-02

**Authors:** Moonsang Lee, Thi Kim Oanh Vu, Kyoung Su Lee, Eun Kyu Kim, Sungsoo Park

**Affiliations:** 1 Korea Basic Science Institute, 169-148, Gwahak-ro, Yuseong-gu, 34133 Daejeon, Korea; 2 Quantum-Function Research Laboratory and Department of Physics, Hanyang University, 04763 Seoul, Korea; vuthikimoanh92@gmail.com (T.K.O.V.); kslee3733@naver.com (K.S.L.); 3 Department of Science Education, Jeonju University, 303 Cheonjam-ro, Wansan-gu, 55069 Jeollabuk-do, Korea; 4 Analytical Laboratory of Advanced Ferroelectric Crystals, Jeonju University, 303 Cheonjam-ro, Wansan-gu, 55069 Jeollabuk-do, Korea

**Keywords:** nanodot, a-plane GaN, HVPE, Schottky diodes

## Abstract

We investigate the electrical characteristics of Schottky contacts for an Au/hydride vapor phase epitaxy (HVPE) a-plane GaN template grown via in situ GaN nanodot formation. Although the Schottky diodes present excellent rectifying characteristics, their Schottky barrier height and ideality factor are highly dependent upon temperature variation. The relationship between the barrier height, ideality factor, and conventional Richardson plot reveals that the Schottky diodes exhibit an inhomogeneous barrier height, attributed to the interface states between the metal and a-plane GaN film and to point defects within the a-plane GaN layers grown via in situ nanodot formation. Also, we confirm that the current transport mechanism of HVPE a-plane GaN Schottky diodes grown via in situ nanodot formation prefers a thermionic field emission model rather than a thermionic emission (TE) one, implying that Poole–Frenkel emission dominates the conduction mechanism over the entire range of measured temperatures. The deep-level transient spectroscopy (DLTS) results prove the presence of noninteracting point-defect-assisted tunneling, which plays an important role in the transport mechanism. These electrical characteristics indicate that this method possesses a great throughput advantage for various applications, compared with Schottky contact to a-plane GaN grown using other methods. We expect that HVPE a-plane GaN Schottky diodes supported by in situ nanodot formation will open further opportunities for the development of nonpolar GaN-based high-performance devices.

## 1. Introduction

Polarization-free nonpolar a-plane GaN layers have been extensively considered as an adequate material for high-performance GaN-based optoelectronic devices owing to the absence of the quantum-confined Stark effect (QCSE) along its orientation [1,2]. Nonpolar GaN-based electronic devices, including high-electron-mobility transistors (HEMT), Schottky diodes, and metal–oxide–semiconductor field-effect transistor (MOSFET) sensors have been investigated to achieve enhanced performance such as enhancement mode (normally-off), temperature-stable characteristics with less pronounced hysteresis, and high sensitivity [3,4,5,6]. Due to the lack of native bulk GaN, researchers have been investigating the growth of a-plane GaN with improved structural properties grown on foreign substrates, using metal organic vapor phase epitaxy (MOCVD), molecular beam epitaxy (MBE), or hydride vapor phase epitaxy (HVPE) [7,8,9,10]. Among these methods, HVPE nonpolar a-plane GaN templates have attracted considerable attention for their high throughput and relatively good crystal quality, and in view of their efficient and effective commercial advantages [11,12]. Nonpolar a-plane GaN layers, however, typically have highly extended defects, such as the densities of threading dislocations and basal, prismatic stacking faults [13]. These structural defects can result in the distortion of the characteristics of metallization contacts. To circumvent the problem, various approaches like epitaxial lateral overgrowth (ELOG), and low-temperature multibuffer layer have been employed [14,15]. These, however, require additional ex situ processes outside the HVPE reactor, which results in low throughput and high cost. Moreover, external processes can cause the evolution of unintentional properties of a-plane GaN layers. Recently, we proposed the simple and efficient method of growing HVPE thick GaN layers using in situ formation of GaN nanodots without any external processes [16]. Although this can be applied to any Al_2_O_3_ substrate regardless of crystal orientation, the electrical properties of contacts between the metal and a-plane GaN template have to be explored for their opto-electrical applications such as light-emitting diodes (LEDs), laser diodes (LDs), and power electronics. To optimize a-plane GaN-based device performance, it is crucial to determine, understand, and identify the electrical properties of contacts between the a-plane GaN layers and metal. In particular, an investigation of current transport, which is related to the behavior of leakage current characteristics, is required to improve the device reliability. Even though some works have reported the barrier and current characteristics between the a-plane GaN and metal contact [17,18], none have investigated the Schottky mechanism of metal and HVPE a-plane GaN templates grown via in situ GaN nanodot formation.

In this paper, we elucidate the electrical characteristics of Schottky diodes with nonpolar HVPE a-plane GaN templates grown via GaN nanodot formation.

## 2. Materials and Methods

**Figure 1:**
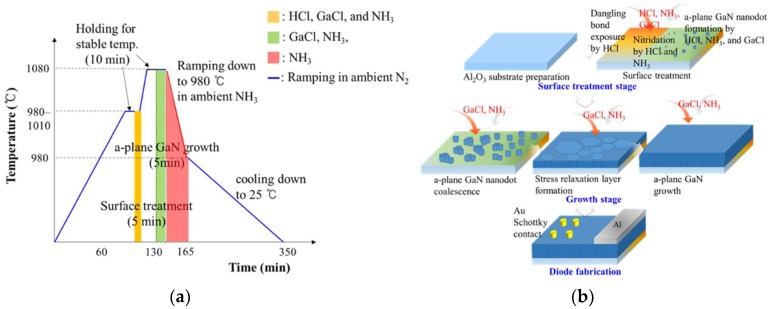
Schematic illustrations of (**a**) the growth sequence of a-plane GaN templates via in situ nanodot formation and (**b**) the fabrication of hydride vapor phase epitaxy (HVPE) a-plane GaN Schottky diodes based on the suggested method.

Nonpolar a-plane GaN layers were grown on 2 inch (1-102) r-plane sapphire substrates (Iljin display, Pyeongtaek, Korea), using a vertical-type homemade HVPE. The configuration of the homemade HVPE system was detailed elsewhere [11]. The stress relaxation layers using GaN nanodots were grown on r-plane sapphire substrates to prevent significant stress evolution during a-plane GaN growth. To achieve this, the substrates were subjected to a surface treatment before nonpolar GaN growth. This was composed of HCl etching (flow rate: 70 sccm) and nitridation with NH_3_ gas (flow rate: 2500 sccm) for 5 min. This treatment resulted in the formation of a discontinuous a-plane GaN nanodot buffer layer on the substrates (see Figure S1). Subsequently, a-plane GaN layers were grown on the a-plane GaN nanodot buffer layer via the reaction between GaCl and NH_3_ under atmospheric pressure and a temperature of ~1080 °C. HCl gas (flow rate: 40 sccm) was reacted with liquid Ga metal to form GaCl gas, which was transported to the growth zone where it reacted with NH_3_. The total flow rate of N_2_ used as the carrier gas was 15,000 sccm and the V/III ratio was approximately 6. Finally, 5 µm thick nonpolar a-plane GaN templates were obtained. Detailed procedures are presented in Figure 1a, and in other literature [16]. The growth kinetics are also explained elsewhere [19]. The densities of stacking fault and threading dislocation were 4.12 × 10^5^/cm, and 3.86 × 10^9^/cm^2^, respectively (not shown in this paper). These values are comparable to those of templates grown by MOCVD [20,21].

For the electrical characterization of HVPE a-plane GaN templates grown via GaN nanodot formation, Schottky diodes were fabricated using Au and Al metallization. First, 150 nm thick and 3 mm diameter Al metal was deposited on the a-plane GaN layers as ohmic contacts using a thermal evaporator (Infinity vacuum, Seoul, Korea). Then, the ohmic contacts were annealed at 550 °C in ambient Ar to improve the ohmic contact formation. Subsequently, Schottky contacts with 300 µm diameters were fabricated by an electron beam evaporator (Sorona, Seoul, Korea) using Au (40 nm) on the a-plane GaN layers, followed by rapid thermal annealing in ambient Ar at 550 °C. The details are presented in Figure 1b.

## 3. Results and Discussion

**Figure 2:**
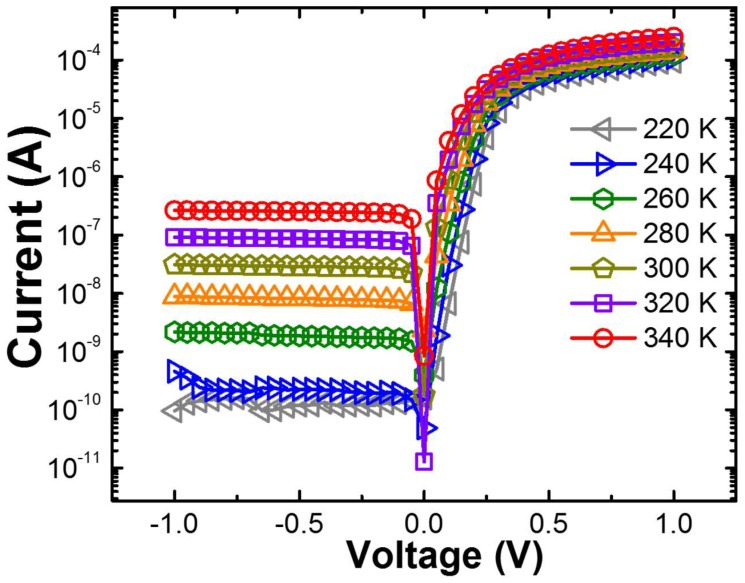
Temperature-dependent *I*–*V* characteristics of Au/a-plane GaN templates grown via GaN nanodot formation.

The forward and reverse current–voltage (*I*–*V*) characteristics of the Schottky diodes formed on a-plane GaN templates grown via GaN nanodot formation in the temperature range from 220 to 340 K are plotted in Figure 2. The forward bias in the current–voltage–temperature (*I*–*V*–*T*) curves exhibits a linear behavior at low forward bias voltages but deviates considerably over 0.4 V from linearity due to the series resistance (R_s_) in the whole temperature range. It is well known that thermally generated current carriers accelerate the larger current flow through the diode [22]. On the other hand, the reverse bias clearly shows excellent rectifying characteristics in the temperature range of 140–220 K, suggesting that Schottky junctions are well formed at the Au/a-plane GaN templates. Even though the rectification characteristics of the diodes decrease over 260 K, the ratios of the forward to reverse current at ±1 V of the Au/a-plane GaN template Schottky diodes in the entire temperature region were from 6.06 × 10^4^ to 1.59 × 10^7^, indicating a good rectification behavior.

The electrical parameters were extracted on the basis of the thermionic emission (TE) theory as follows [23,24]:

(1) $$I=AA^{**}T^{2}\exp\left(-\frac{q\Phi_{B}}{kT}\right)\left[\exp\left(\frac{qV}{nkT}\right)-1\right]\;\mathrm{for}\;V\;\geq \;3kT/q$$

(2) $$I_{0}=AA^{**}T^{2}\exp\left(-\frac{q\Phi_{B}}{kT}\right)$$

where *I*_0_, *A*, A^**^, *k*, *T*, *q*, *n*, *Φ_B_*, and *V* represent the saturation current, contact area, Richardson constant (26.4 A·cm^−2^·K^−2^ for n-type GaN), Boltzmann constant, temperature, electron charge, ideality factor, Schottky barrier height, and applied voltage, respectively.

**Figure 3:**
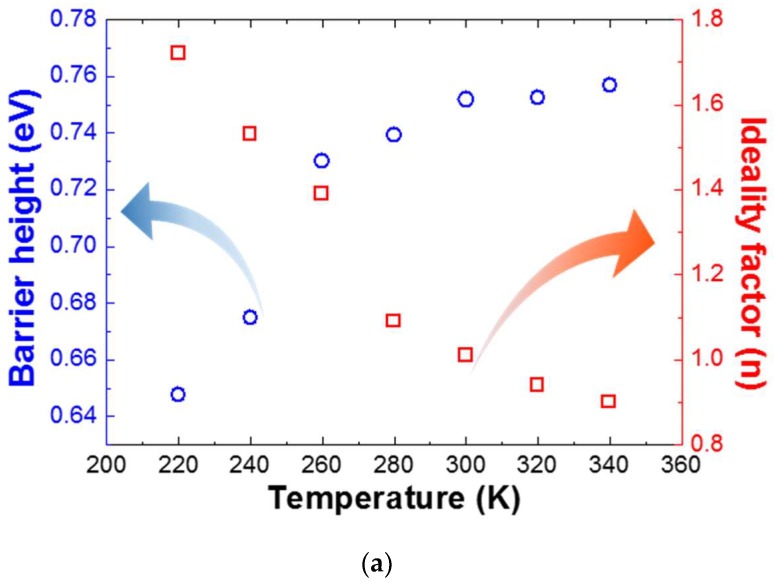

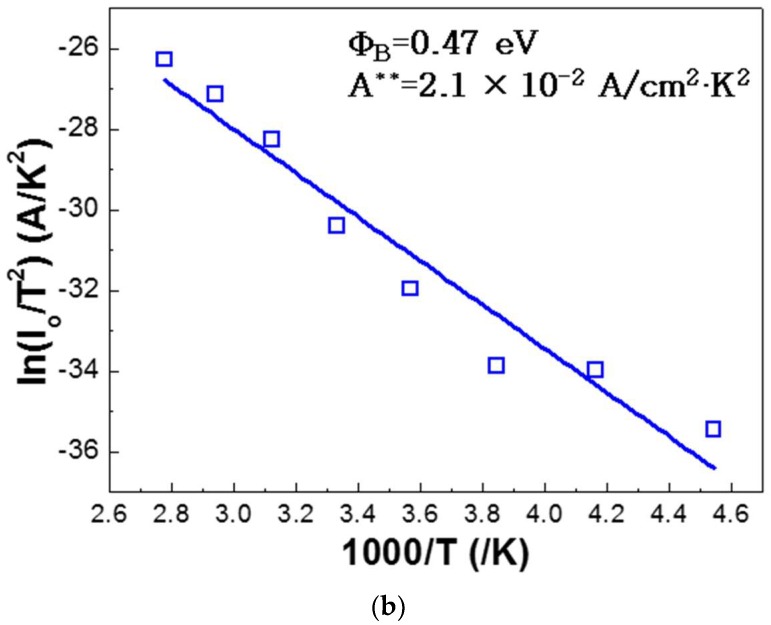
(**a**) Barrier height and ideality factor versus temperature in Au/HVPE a-plane GaN templates formed by in situ nanodot formation; (**b**) Richardson plot of ln(*I*_0_/*T*^2^) versus 1000/*T* for Au/HVPE a-plane GaN Schottky contact.

The measured barrier heights and ideality factors are listed in Figure 3a. It is worth noting that at ~260 K, the gradient of the barrier height and ideality factor significantly changed with the temperature gradient. This is a commonly observed phenomenon in Schottky diodes, and is attributed to the inhomogeneity of the barrier height in the contact [25]. Taking into consideration the current transport across the metal/semiconductor interface with a temperature-activated process, the dominant electrons flowing at low temperature exist at the lower Schottky barrier height because they cannot surmount the higher Schottky barrier height. However, at high temperature, electrons with sufficient energy can overcome the high Schottky barrier height, thereby increasing the dominant barrier height [26]. When applying the TE model, the ideality factors are varied from 0.88 to 1.74 in the temperature range of 220–340 K. From the slope of the Richardson plot, the barrier height corresponding to the activation energy was obtained in the temperature range from 220 K to 340 K, as seen in Figure 3b. We extracted 0.47 eV, a barrier height of 2.1 × 10^−2^ A/cm^2^·K^2^ and the Richardson constant from the linear fit to the plot. It is interesting to note that the *Φ_B_* obtained from the figure is much smaller than the value of the TE model. The Richardson constant deviated by a decrease of almost three orders of magnitude from the theoretical value of 26.4 A/cm^2^·K^2^ for n-GaN. This can be explained by the spatially fluctuated barrier height in the contacts between the Au metal and a-plane GaN templates grown via GaN nanodot formation [27,28]. These results clearly suggest the formation of an inhomogeneity of *Φ_B_* on the Au/a-plane GaN templates grown via GaN nanodot formation, which can be attributed to the surface defects in nonpolar a-plane GaN [29,30,31].

It is essential to note that the higher deviation of ideality factors from unity at low temperature, even including inhomogeneous Schottky barrier heights, indicate that TE is not the only mechanism for the current transport. Note that the value of *n* is the measure of the extent to which the properties of a diode resemble the ideal diode characteristics and reflect the current transport mechanism in a device [32,33]. Other conduction mechanisms, such as thermionic field emission (TFE), field emission (FE), multistep tunneling via interface state, or dislocations, can be alternatives [34]. Considering that the TFE dominates the charge transport with a deviated ideality factor, the transport mechanism is given by [35]

(3) $$I=I_{0}\exp\left(\frac{qV}{E_{00}\coth\left(E_{00}/kT\right)}\right)=I_{0}\exp\left(\frac{qV}{E_{0}}\right)$$

(4) $$I_{0}=\frac{AA^{**}T\sqrt{\pi E_{00}q\left(\Phi_{B}-V-\xi \right)}}{kcosh\left(E_{00}/kT\right)}\times \exp\left(-\frac{q\xi }{kT}-\frac{q\left(\Phi_{B}-\xi \right)}{E_{00}\coth\left(E_{00}/kT\right)}\right)$$

where *E*_00_ = (*qħ*/2)(*N_D_*/m^*^ɛ_s_)^1/2^ is the characteristic energy related to the tunneling probability of potential barrier; *E*_0_ = *E*_00_coth(*E*_00_/*kT*); *V* is the applied bias voltage; *ξ* is equal to E_C_−E_F_, corresponding to *k*T/qln(*N_C_/N_D_*), where N_C_ is the effective density of states in the conduction band (*N_C_* = 2.53 × 10^18^ cm^−3^ in GaN) [36]; *h* is Planck’s constant; m^*^ is the effective mass; and *ɛ_s_* is the dielectric constant.

The Schottky barrier heights and ideality factors in the temperature range from 220 to 340 K were estimated to be 0.87–1.48 eV and 0.88–1.34 respectively. (See Figure S2) Note that the ideality factors obtained from the TFE model are closer to unity, compared with those from the TE model. This verifies that the TFE, namely, Poole–Frenkel emission, is more appropriate to explain the current transport mechanism of HVPE a-plane GaN Schottky diodes formed through in situ nanodot formation over the entire range of measured temperatures. This behaviour is not different from the transport process of Schottky contacts to c- and a-plane GaN grown using other methods [17,18,37]. In addition, the Schottky barrier characteristics are comparable to those using the same contact material and similar diode fabrication conditions [38,39].

**Figure 4:**
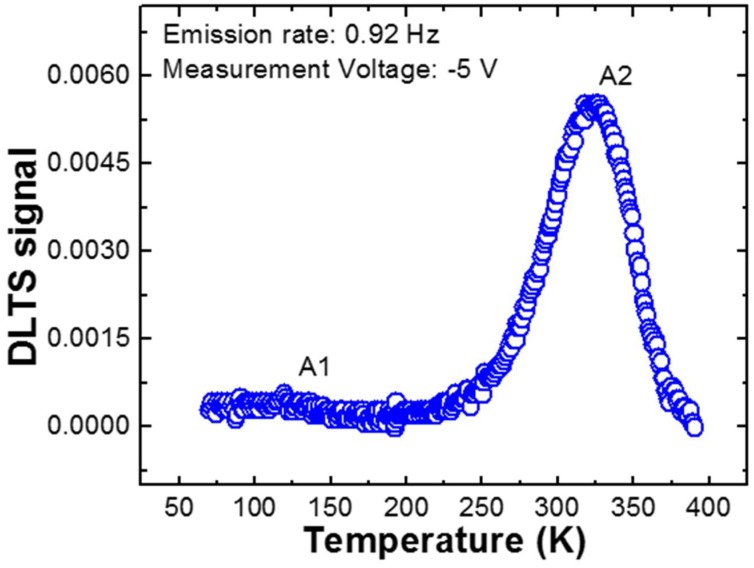
Deep-level transient spectroscopy (DLTS) spectrum of Au/HVPE a-plane GaN template Schottky diodes grown via in situ nanodot formation, measured at a reverse voltage of −5 V in the temperature range of 70–370 K.

Even though the TFE model explains the current transport mechanism of HVPE a-plane GaN grown via nanodot formation well, one can observe the slight deviation of ideality factors from unity. Obviously, tunneling and high series resistance in the high voltage region can result in the inhomogeneity of *Φ_B_*. Also, the interface states are responsible for band bending, and behave as trap-assisted tunneling sites, giving rise to an increased ideality factor [34,40]. In addition, it is well known that HVPE a-plane GaN layers are prone to the formation of nitrogen desorption, nitrogen-related surface states, complex defects with V_Ga_ such as V_N_-V_Ga_, and nitrogen anti-sites (N_Ga_) during growth [41,42]. This encourages the tunneling process through the barrier, thus distorting the ideal Schottky characteristics. To elucidate the effects on unintentional charge traps, we performed deep-level transient spectroscopy (DLTS) measurements at a reverse voltage of 5 V in the temperature range of 70–370 K, as shown in Figure 4. We can clearly observe the two deep trap sites from the plateau. These trap levels exist at ~0.2 eV and ~0.55 eV below the conduction band edge. These are related to the noninteracting point defects [41,43,44,45]. These electronic deep trap carriers were filled at low temperature and released at over 260 K, thus increasing *ϕ_B_*. Note that the DLTS signal abruptly starts to increase around 260 K. Therefore, we are assured that deep-level defects within the a-plane GaN template grown via in situ nanodot formation can be one of the important sources causing variation in *Φ_B_* and *n* for the two linear fitting regions around 260 K, confirmed by DLTS at different energy levels below the conduction band edge as shown in Figure 4. It is noticeable that the characteristics of the unintentional charge traps embedded in the a-plane GaN template grown via in situ nanodot formation are comparable to those of a-plane GaN layers grown using other methods [46,47]. This indicates that the a-plane GaN templates grown via in situ nanodot formation possess a great throughput advantage for various applications, compared to those grown using other methods. Table 1 summarizes the activation energy, capture cross section, and trap density obtained by DLTS analysis.

## 4. Conclusions

*I–V–T* measurements, conduction models, and DLTS analysis have been employed to investigate the electrical characteristics of Schottky diodes fabricated on HVPE a-plane GaN templates grown using in situ GaN nanodot formation. The barrier height and ideality factors are highly dependent upon the temperature gradient. Deviated ideality factors from unity reveal the inhomogeneity of the barrier height, which was confirmed by conventional Richardson, barrier height, and ideality plots. Also, we confirmed that TFE conduction is more appropriate to explain the current transport mechanism of HVPE a-plane GaN Schottky diodes grown via in situ nanodot formation, compared with the TE model. Furthermore, DLTS measurements suggest that defect-assisted tunneling played an important role in the Au/HVPE a-plane GaN Schottky contacts due to the presence of noninteracting point defects. The electrical characteristics of a-plane GaN templates grown using in situ nanodot formation are comparable to those of Schottky contacts to a-plane GaN layers grown using other methods. We believe that the formation of Au/HVPE a-plane GaN Schottky diodes based on in situ nanodot growth is promising for the application of nonpolar GaN-based opto-electrical devices.

## Figures and Tables

**Table 1:** Defect parameters for Au/a-plane GaN Schottky diodes.

Ref.	Defect	Activation Energy (eV)	Capture Cross Section (cm^2^)	Trap Density (cm^−3^)
This study	A1	0.20	1.14 × 10^−17^	5 × 10^12^
	A2	0.55	4.4 × 10^−17^	4.7 × 10^13^
[42]	E1	0.23	5.43 × 10^−15^	9.5 × 10^13^
	E2	0.5	4.65 × 10^−17^	2.3 × 10^14^
[45]	EA1	0.56	9.72 × 10^−16^	3 × 10^13^
[46]	E3	0.58	8.9 × 10^−12^	3.8 × 10^14^

## Supplementary Materials

The following are available online at http://www.mdpi.com/2079-4991/8/6/397/s1. Figure S1: Plan-view SEM image of a-plane GaN nanodots formed on r-plane sapphire substrate after in situ surface treatment. Figure S2: Barrier height and ideality factor versus temperature in Au/HVPE a-plane GaN templates formed by in situ nanodot formation based on TFE model.
