# Supplementary material for: Electronic Transport Mechanism for Schottky Diodes Formed by Au/HVPE a-Plane GaN Templates Grown via In Situ GaN Nanodot Formation
Source: Nanomaterials (Basel). 2018 Jun 2;8(6):397. doi: 10.3390/nano8060397 (PMC6027380; doi:10.3390/nano8060397)
Supplement: Supplementary file 1 [file nanomaterials-08-00397-s001.pdf]

Supplementary Materials

Electronic transport mechanism for Schottky diodes formed by Au/HVPE a-plane GaN templates grown via in situ GaN nanodot formation

Moonsang Lee [1,*], Thi Kim Oanh Vu[2], Kyoung Su Lee[2], Eun Kyu Kim[2,*], and Sungsoo Park[3, 4, *]

[1] Korea Basic Science Institute, 169-148, Gwahak-ro, Yuseong-gu, Daejeon, Republic of Korea; lms1015@kbsi.re.kr

[2] Quantum-Function Research Laboratory and Department of Physics, Hanyang University, Seoul 04763, Republic of Korea; e-mail@e-mail.com

[3] Department of Science Education, Jeonju University, 303 Cheonjam-ro, Wansan-gu, Jeollabuk-do, Republic of Korea; sspark@jj.ac.kr

[4] Analytical Laboratory of Advanced Ferroelectric Crystals, Jeonju University, 303 Cheonjam-ro, Wansan-gu, Jeollabuk-do, Republic of Korea

* Correspondence: lms1015@kbsi.re.kr, ek-kim@hanyang.ac.kr, and sspark@jj.ac.kr; Tel.: +82-42-865-3519(M. Lee)

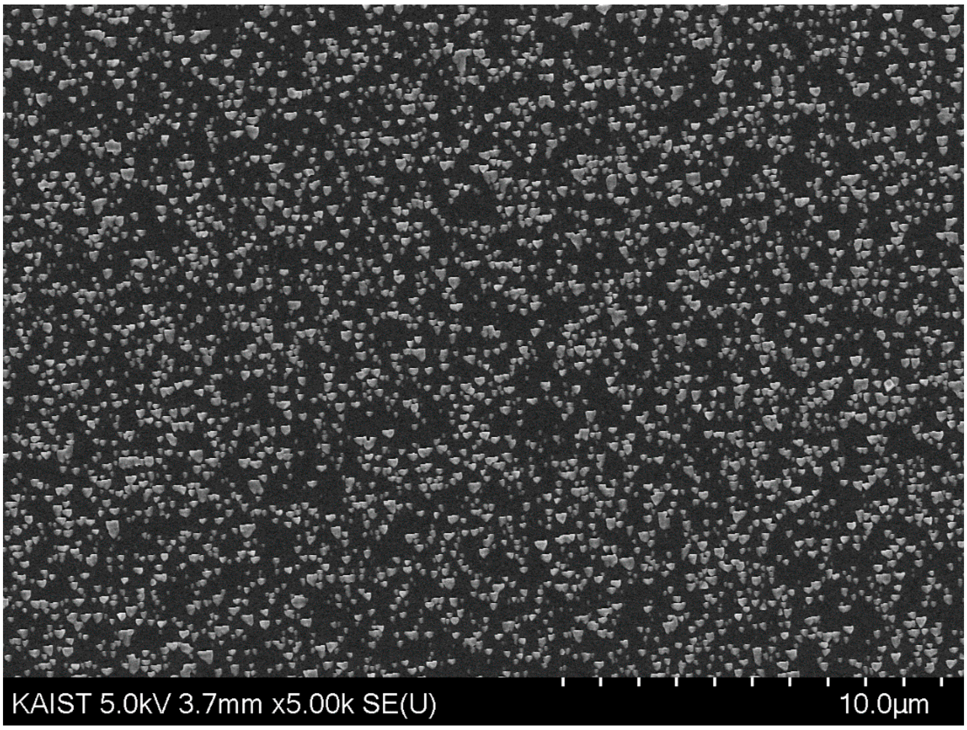

Fig. S1 Plan-view SEM image of a-plane GaN nanodot formed on r-plane sapphire substrate after *in situ* surface treatment.

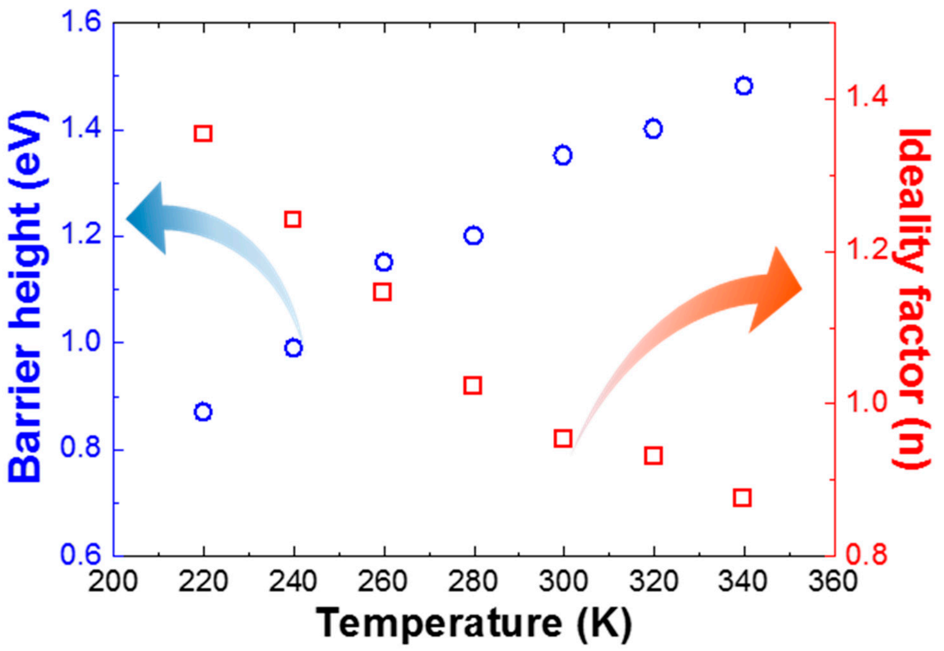

Fig. S2 Barrier height and ideality factor versus temperature in Au/HVPE a-plane GaN templates formed by in situ nanodot formation based on TFE model.
